# Enhanced community reintegration: the impact of participating in wheelchair basketball on reintegration of people living with a spinal cord injury

**DOI:** 10.3389/fspor.2026.1742238

**Published:** 2026-06-17

**Authors:** Nothemba Thobela, Lieketseng Ned

**Affiliations:** Division of Disability and Rehabilitation Studies, Department of Global Health, Stellenbosch University, Stellenbosch, South Africa

**Keywords:** basketball, community, rehabilitation, reintegration, spinal cord injury, wheelchair

## Abstract

**Introduction:**

Spinal cord injury (SCI) is a life-altering condition with significant physical, psychological, and social consequences. In South Africa, where trauma-related SCI is prevalent and specialized rehabilitation services are limited, community reintegration remains a major challenge. Participation in adaptive sports, such as wheelchair basketball, may provide opportunities for improved independence, social support, and psychosocial well-being. Therefore, this study aimed to explore the role wheelchair basketball plays to support community reintegration of people with SCI into their community within the Western Cape province.

**Methods:**

A qualitative, ethnographic design with a mixed-methods approach was used to gather in-depth information on the lived experiences of wheelchair basketball players living with SCI. Four participants (2 males and 2 females, aged 19–26 years) were recruited from a local wheelchair basketball club in the Western Cape, South Africa. A noted significant limitation of the study was the limited number of participants due to the focus on one health condition. Post-COVID19, many of the wheelchair basketball clubs in the region were unable to fully function again, therefore, there was only one local club that could provide suitable participants. Therefore, as an in-depth ethnographic exploration of lived experiences within a single wheelchair basketball club, the sample size was appropriate for capturing rich, contextual data. Data was collected using the Community Integration Questionnaire (CIQ), the Community Integration Measure (CIM), and semi-structured interviews. Quantitative data were analyzed descriptively, while qualitative data underwent thematic analysis following Braun and Clarke's six-step framework.

**Findings:**

Thematic analysis revealed three main themes: improved functional independence, improved inclusivity and socialization, and enhanced self-esteem and self-confidence. Questionnaire findings showed higher perceived integration than objectively measured integration, with social engagement emerging as a major facilitator of reintegration.

**Discussion:**

Participation in wheelchair basketball facilitated physical, social, and psychological aspects of community reintegration. Despite systemic barriers, the sport offered valuable opportunities for independence, peer support, and empowerment. The discrepancy between perceived and objective integration suggests wheelchair basketball may enhance subjective belonging even when structural barriers persist. Broader support from local and national stakeholders is needed to sustain these benefits.

**Conclusion:**

Wheelchair basketball can serve as a valuable tool for enhancing community reintegration among individuals with SCI, though greater institutional and financial support is required. Larger studies are needed to confirm these findings across diverse contexts.

## 1 Introduction

Spinal cord injury (SCI) is a severe, life-altering injury causing permanent disability, with the global incidence increasing significantly from 2013 to 2019, reaching an estimated 900,000 cases. This rise is attributed to increased global life expectancy and the subsequent rise in Years Lived with a Disability (YLD) (1). While falls and road injuries are common causes globally, South Africa faces a unique challenge with 60% of SCI cases resulting from trauma, predominantly violence (gunshot and stab wounds), reflecting the country's ongoing socio-political issues and violence (2). This trauma-driven SCI incidence exacerbates South Africa's existing quadruple burden of disease, highlighting the urgent need for effective and sustainable management strategies (3).

Community reintegration, defined as the restoration of individuals to active participation in family and community life (4), therefore, becomes crucial for optimizing quality of life and functional independence following SCI. Community-Based Rehabilitation (CBR), introduced in Southern Africa in 2000, aims to address this gap by improving access to rehabilitation services and promoting social integration (5, 18). Despite its potential, the implementation of CBR faces challenges, particularly in resource-limited and rural settings. Therefore, a more comprehensive approach was required to bridge the gap between hospital-based rehabilitation and successful community reintegration for individuals with SCI in South Africa.

The SCI community is at high risk for sedentary lifestyles, increased physical activity can decrease secondary health problems like obesity and metabolic disorders (4). It also combats social isolation and improves overall quality of life (4). Although factors such as pre-morbid sports participation, exposure to wheelchair sports, access to sporting teams, level of injury, and fear of re-injury may influence reintegration, the communal and psychosocial benefits associated with team sports may still be experienced in the absence of direct participation (19). By being a part of the communal and social environment they foster, it can promote a sense of belonging, social inclusion and connectedness, and create an environment for psychosocial and emotional support (19). Wheelchair basketball is therefore, highlighted as a valuable tool for building social support among people with SCI because it is a team sport. Team sports foster camaraderie and peer support, helping individuals with an SCI overcome social isolation and negative self-perception often caused by stigma (20). However, there are barriers to participation which include accessibility, financial costs, co-morbidities, and lack of motivation or social support (4). Conversely, facilitators include improved health, reduced complications, increased peer support, better quality of life, and enhanced self-efficacy, confidence, and reliance (4). Ultimately, participation in organized activities like wheelchair basketball can be considered a comprehensive approach to bridge the gap between hospital-based rehabilitation and community reintegration.

Despite growing evidence supporting adaptive sports participation for individuals with spinal cord injury, limited research has examined the specific mechanisms through which wheelchair basketball facilitates community reintegration, particularly in low-resource contexts like South Africa. Furthermore, few studies have combined quantitative measures of integration with qualitative exploration of lived experiences to understand both objective outcomes and subjective perceptions.

Therefore, the aim of this study was to explore the role wheelchair basketball plays to support community re integration of people with SCI into their community within the western cape province. This would be achieved by:

- Exploring how participating in wheelchair basketball can be utilized as transitional medium to facilitate continuous rehabilitation in the community for people with SCI.
- Exploring the social support systems and self-efficacy provided through participating in wheelchair basketball for people with SCI.
- Determining how wheelchair basketball for people with SCI can enhance re-integration and participation into their community.

By addressing these objectives, this study contributes to understanding how adaptive sports can bridge the gap between institutional rehabilitation and successful community reintegration for individuals with spinal cord injury in South Africa.”

## 2 Materials and methods

### 2.1 Study design

Overall, a qualitative, interpretivist approach was used, utilizing ethnography as the research design. Ethnography was selected as it focuses on the study of a group of people through immersion of the investigator within the culture of that group for an extended period (21). The aim of ethnography is to understand the behavior or culture of the group through the perspective of the members of the group (21). The goal was to understand the behaviors and culture of the Western Cape wheelchair basketball community from the team members' perspectives through shared experiences, peer learning, and cultural practices around disability. To achieve this, a mixed-methods approach was adopted by primarily using qualitative methods as a data collection strategy to capture rich, subjective insights into community reintegration, supplemented by quantitative tools to provide objective measurements and enable triangulation of findings.

### 2.2 Ethical approval statement

The study involving humans received ethical approval by the Health Research Ethic Committee (Reference: SS22/04/057) at the University of Stellenbosch, Faculty of Medicine and Health Sciences Centre for Disability & Rehabilitation Studies. The studies were conducted in accordance with local legislation and institutional requirements. Written informed consent for participation in this study was obtained from all participants, as all were adults aged 18 years or older. No legal guardians or next of kin were involved in consent procedures.

### 2.3 Data collection methods and process

The study focused on members of the Conquerors wheelchair basketball club in the Western Cape, South Africa. The participants were recruited purposive sampling from the club, targeting individuals who have sustained both traumatic and non-traumatic SCI. This study specifically focused on those with a SCI because people with a SCI have the least access and support, especially for community integration, and they remain the most physically inactive.

#### 2.3.1 Inclusion and exclusion criteria

Inclusion criteria were established to ensure participants had sufficient community and team experience to reflect on reintegration processes. It specified individuals with SCI who:

- Have resided in the same community for over 6 months
- Been a team player for over 3 months
- 18 years or older
- Having an SCI for over 3 months
- Having gone through the physical rehabilitation process
- Living in a productive community and
- Having a basic understanding of the English language.

Exclusion criteria were applied to avoid confounding factors and these included:

- Having any disability other than SCI
- Residing in the same community for less than 6 months
- Basketball team participation for less than 3 months
- Younger than 18 years of age
- Sustained a SCI for less than 3 months
- Not having gone through any form of the rehabilitation process at all
- Living in any form of institutionalized setting
- Inability to read and understand a basic level of English.

#### 2.3.2 Study population and sampling

Unlike quantitative research, sampling in qualitative research is deliberate and not random (22). Malterud et al. (23), proposed that a sample size with sufficient information power will depend on; aim, specificity, established theory, quality of dialogue and analysis strategy. Sufficient information power was determined by reaching the point of data saturation, which is a point in data collection when new data no longer brings additional insights to the research questions and no longer bring emerging new themes (24). Therefore, the more informative the sample is, the lower the number of participants required to reach saturation (23). The main strategy of sampling for an ethnographic study is the use of purposive sampling.

Purposive sampling is sampling that is done on a group of participants that are chosen from a preselected criterion that is relevant to a particular research question (25). Sample sizes, which may or may not be fixed prior to data collection, depend on the resources and time available, as well as the study's objectives and they are often determined based on data saturation, and this was applied in this study.

A wheelchair basketball team consists of players with a range of varying disabilities and very limited in size. The inclusion/exclusion criteria further limited it to people with SCI which reduced the population significantly. Of the 40 active players in the club, there were an estimated 18 participants with a SCI who were eligible to partake in the study. Once the inclusion/exclusion criteria were applied (in combination with who was willing to participate), the number of participants significantly reduced to a 4–10 participant range (see Table 1 for participants demographics).

**Table 1:** Participant demographics.

Participants	Age	Gender	Race	Occupation	Medical diagnosis
AX1000	20	Female	African	Learner ship Programme	T9 Traumatic spinal cord injury
NK2000	22	Female	African	University Student	Spina Bifida
MM2000	19	Male	African	Unemployed	Spina Bifida
SM1000	26	Male	African	University Student	T2 Traumatic spinal cord injury

#### 2.3.3 Data collection tools

Multiple methods of data collection were used including questionnaires and semi-structured interviews.

##### 2.3.3.1 Questionnaires

The questionnaires were used as a tool to assess the level of community reintegration and these were namely the; the Community Integration Questionnaire (CIQ) and the Community Integration Measure (CIM).

The Community Integration Questionnaire is a validated 15-item inventory designed to measure levels of community integration compromising three subscales: home integration, social integration, and productivity (6). Although it is primarily a qualitative measure of community integration, it possesses several quantitative aspects (6). The total scores range from 0 to 29, with higher scores indicating greater integration. The CIQ has demonstrated good test-retest reliability and internal consistency in populations with neurological conditions.

The Community Integration Measure (CIM) is a 10-item, client-centered measure based on the ICF's participation domain. It uses the words of participants themselves and makes no assumptions about the relative importance of specific activities or relationships (7). Each item is rated on a 5-point Likert scale, with total scores ranging from 10 to 50. Higher scores reflect greater perceived integration. The CIM has demonstrated strong internal consistency and construct validity in rehabilitation populations. The CIM is purely qualitative and when used in conjunction with the CIQ, it increases the validity and depth of the information being gathered from different/multiple perspectives (7).

##### 2.3.3.2 Semi-structured interviews

Semi-structured interviews, guided by a pre-set interview protocol and guide, were conducted to explore participants' experiences and perspectives. This guide consisted of open-ended questions with probes that were designed to relate to the study objectives and ultimately the aim of the study. Further probing and clarifying questions were asked when necessary. Interviews were conducted virtually and face-to-face, depending on participant preference and circumstances. All interviews were conducted and completed by the researcher in the participants' language of choice which were either isiXhosa, English or a mix of both languages in cases where further clarification was required. Each interview lasted approximately 20–30 min. The interview sessions were recorded using a phone recorder and recording options on platforms such as Zoom with verbal permission of the participant. Notes were also taken for clarity in case of misunderstandings and poor connectivity over the virtual platform. Due to the nature of the study being conducted virtually, participants were provided with data vouchers to assist with internet access to complete their questionnaires and participate in the follow-up virtual interviews for more narrative data. For those who were unable to complete the questionnaires online, they were provided with a stipend that assisted with printing and/or scanning needs.

### 2.4 Data management

Collected data, including questionnaires and interview recordings, were stored securely on password-protected computers and cloud storage. Interviews were transcribed verbatim and the transcripts were de-identified to ensure participant confidentiality. All data was kept confidential in accordance with Stellenbosch University Policy for Responsible Research Conduct (26) and Stellenbosch University Records Management Policy (27).

### 2.5 Data analysis

Quantitative data from the questionnaires were analyzed using descriptive statistics. Qualitative data from the interviews were analyzed using inductive thematic analysis, following Braun and Clarke's (8) six-step framework:

Step 1: Familiarization—Transcribing the data and repetitive listening to the audio recordings followed by rereading the data for clarity thereby picking up any similarities to become more familiar with the data.
Step 2: Generation of initial codes—Once the data was transcribed, the interview guide questions were noted and highlighted and similar answers from each transcription were grouped to the relevant question. The data were tabulated, and codes were generated from the table.
Step 3: Searching for themes—From the above coded data, potential themes were extracted.
Step 4: Reviewing themes—Once the potential themes were identified, they were checked to see if they were in line with the coded data and the overall data set. This required more in-depth studying of the data set and potential themes by re-reading and reviewing it until data saturation was reached and no new themes were emerging from the data.
Step 5: Defining and naming themes—Once the potential themes were identified, analysis continued to define simple terms for the themes.
Step 6: Writing the report—Analysis was completed in conjunction with the research questions and the findings discussed.

Analysis and transcription of the recordings allowed a manuscript to be produced from which categories or codes could be reviewed and grouped into potential themes and then analyzed for emerging themes. Concurrently, the questionnaires were scored and the results compared to the emerging themes from the interview data to determine if there were any similarities between what the themes suggested and the participants' perceived level of community re-integration when scoring themselves. These themes were then reviewed and restructured to be in line with the study aims and objectives.

### 2.6 Trustworthiness

Trustworthiness was addressed through criteria including credibility, transferability, dependability, and confirmability, as described by Guba and Lincoln (9). Credibility was enhanced through the use of standardized tools and member checking.

## 3 Findings

### 3.1 Questionnaire findings

Four participants completed both questionnaires. CIM scores ranged from 35 to 41 out of 50, representing 70–82 per cent of the maximum possible score, with a mean of 38. This is indicative of a moderate to high level of integration (see Table 2).

**Table 2:** CIM and CIQ scores.

					
	CIM	CIQ			
Participants’ codes	Total	Home	Social	Productive	Total
AX1000	39/50	4/10	7/12	3/7	14/29
NK2000	41/50	4/10	5/12	6/7	15/29
MM2000	37/50	3/10	9/12	2/7	12/29
SM1000	35/50	1/10	10/12	3/7	14/29

Total CIQ scores ranged from 12 to 15 out of 29, representing 41–52 per cent of the maximum possible score, with a mean of 13.8. This is indicative of mild to moderate level of integration.

Subscale analysis revealed home integration scores ranging from 1 to 4 out of 10, with a mean of 3; social integration scores ranging from 5 to 10 out of 12, with a mean of 7.8; and productivity scores ranging from 2 to 6 out of 7, with a mean of 3.5.

Three participants scored below average on home and productivity subscales, while all participants scored at or above the midpoint on the social subscale. This indicated that they are not fully integrated into their community if only one of the subscales is above average. Once we started looking at the themes from the interview transcripts and findings, we started to notice why the home and productivity sub-scales score were so low in the CIQ.

### 3.2 Interview findings

This section presentedthe themes and the sub-themes that emerged from the interviews conducted with the four participants. Three main themes (with sub-themes) emerged from the study data and were in line with the objectives of the study (see Table 3).

**Table 3:** Emerging themes in relation to study objectives.

Objectives addressed by the theme	Themes	Sub-themes
How participating in wheelchair basketball can be utilized as transitional medium to facilitate continuous rehabilitation in the community for people with SCI.	Theme 1: Improved functional independence	▪ Perceived community integration level ▪ Decreased need for physical assistance ▪ Improved accessibility ▪ Decreased secondary complications.
Exploring the social support systems and self-efficacy provided through participating in wheelchair basketball for people with SCI.	Theme 2: Improved inclusivity and socialization	▪ Peer support ▪ More travelling opportunities and social outing ▪ Exposure to productive participation
To determine how wheelchair basketball for people living with a SCI can enhance re-integration and participation into their community.	Theme 3: Improved self-esteem and self-confidence	▪ Decreased self-isolation and improved sense of purpose. ▪ Community role model ▪ Improved social support from friends and family.

Theme 1: Improved functional independence

This theme focused on how participation in wheelchair basketball had aided with improving functional independence which resulted in participants requiring less physical assistance with their activities of daily living and accessing their community. They also showed a reduced risk of developing secondary complications. This overall translated into improved functional independence at home and in their community.

Sub-theme A: Decreased need for physical assistance

Participants described that by constantly being physically active, they were able to mobilize around the obstacles in their community more easily because they were physically fit to do so. And by being exposed to other wheelchair users, they also learnt tips and tricks on how to navigate home and community barriers independently, mostly from the older generation who now live independently.

“..it was my first time travelling and travelling with other wheelchair users and we had to do everything ourselves as a team without asking for help.”—SM1000

“So, I don’t look at it as just a sport but as a way of learning how I can do other things and what I can do while in my wheelchair.”—SM1000

“I think it would be using public transport because taxi drivers don’t want to stop for us because they think that we can’t carry ourselves to get in the car, they think that we will be a burden to lift in the car meanwhile we don’t even need their help.”—AX1000

Sub-theme B: Improved accessibility

Participants felt that even though their immediate environment was not wheelchair accessible, they have had to learn to perfect their wheelchair skills and learn to navigate their environment to make it accessible to them. The participants also explained that by participating in wheelchair basketball, they have been made to face barriers that they would not have been exposed to or willingly tried to face e.g., boarding a bus/plane/taxi, going to the mall or stadium, accessing multi-storey buildings, especially now in times of load reduction where elevators or escalators do not work, etc.

“My first time I went on a bus was with basketball on our way to Johannesburg, and that was the first time I used a bus since my injury”—SM1000

“I think since I'm growing, I find things more flexible and I think I am more independent because I can even go to the mall alone and help myself in every challenge that I meet in the road. I can still help myself.”—MM1000

Sub-theme C: Decreased secondary complications

Three out of the four participants reported a history of secondary complications. Two of the participants had secondary complications within the first two years post-injury and no re-occurrences since then. Only one of the participants reported a history of recurrent pressure sores and hospitalization. The participant that reported the most secondary complications is a part time wheelchair user and they have the ability to walk assisted for short distances.

“I have only ever had an operation on my right leg because I had a bed sore on my bum after my injury”—MM2000

“After I got the spinal operation in December 2021, I have only ever gone for bladder stone crushing surgery”—SM1000

Only one of the participants reported a history of recurrent pressure sores and hospitalization. The participant that reported the most secondary complications is a part time wheelchair user and they have the ability to walk assisted short distances.

Theme 2: Improved inclusivity and socialization

This theme evaluates how being part of a wheelchair basketball team allowed the participants to be part of a supportive community of peers. It focused on improved inclusivity and socialization through participation in wheelchair basketball which was achieved through formation of peer support relationships and participation in productive activity within their communities. It also allowed for more travelling opportunities and social outings that in turn improved peer support and further exposure for productive activities. This decreased the level of self-isolation and opened opportunities to gain more skills and self-confidence.

Sub-theme A: Peer Support

People living with a SCI tend to feel they are the only ones facing a particular problem, until they meet people with similar problems. This is when they discover they have shared problems with potential shared solutions. Participants expressed an element of psychosocial support from their teammates and management staff and in some cases, the support they received the most of in their lives came from members of the basketball team rather than their friends or family. This support brings about a certain level of empowerment amongst the group that often expands outside of the sport itself.

“It gave me a motive that there's..uhm.. that I can put myself in that place that I want to be like the others, you see. That I’m not the only one that used to struggle at certain places but now that we part of a team together, we can still do whatever we wanna do if we are together. And then it teaches me how to communicate to other people and so on.”—AX1000

“Before I played basketball, I think I felt insecure about myself and unwelcomed like I was scared to be around people. Another thing is I was not in a special school, I was in a mainstream school so there were no disabled people at the mainstream school. So, after I went to a special school, I joined basketball and then after I joined, I realized there is nothing that can stop me to not participate in sport, I realized there actually more opportunities for people with disabilities.”—MM2000

“When it comes to friends, the friend I have are the friends I met when I started playing basketball, I don’t have any other friend really either than those ones I play basketball with.”—SM1000

“That was my first-time watching people play basketball and now I'm trying it. I got a chance to meet a lot of people and being around other wheelchair users, you feel freer compared to being around people who don’t use wheelchairs. There are a lot of things I learned from the people in the basketball community, because you get that most of them have been wheelchair users for over 15–20 years and they live alone in their homes and not with other people, so yeah my life has changed a lot. And like now I know how to transfer into the bathtub, something I have never done before because I was scared of doing it until now when I met these people.”—SM1000

“..since I'm on first year but I think it was a little bit difficult for me because I didn't know that, for example, there's the psychology building, I didn’t know there were ramps that could take me to the rooms so every day, I think for a full term, I didn’t attend classes and I was doing it online because I could not access it. Until I met this other girl who uses a wheelchair in the second year and she told me there are lifts behind the building but they are very far.”—NK2000

Sub-theme B: More travelling opportunities and social outings

One of the biggest opportunities that being part of a local wheelchair basketball club provided was the opportunity to represent your province on the national stage and much travelling was required to participate in the inter-provincial tournaments. This travelling came in various forms such as, by public taxis and busses, or by airplanes with the assistance of the management staff or people who have been wheelchair users for a long period of time. Most of the participants expressed that their first time travelling long distance outside of the Western Cape Province was because of wheelchair basketball. Some of the participants said that the travelling opportunity helped them to be more comfortable with using their own local public transport independently in their communities which in turn increased their participation in social outings around their community. They were not anxious to use local transport anymore once they had gained the skills to do it independently and safely with minimal to no assistance and most importantly, the confidence.

“..It's almost like I'm their leader in the community because I have travelled more unlike them.”—NK2000

“..they have done something for me just by being out of the country and knowing some places and people and knowing how to play different and playing different teams.”—MM2000

“..now that I play wheelchair basketball because there are a lot of things, I’ve learned to do like last year we went to Johannesburg twice and it was my first-time travelling and travelling with other wheelchair users. My first time I went on a bus since being in a wheelchair was with basketball on our way to JHB, that was the first time I used a bus since my injury, it was something I’ve never done before”—SM1000 “I think it's better, it's the same as before because now I go outside more, I don’t stay inside like I used too in 2017/2018. It's really not the same, there's been a big difference”—SM1000

Sub-theme C: Exposure to productive participation

The participants felt like they were exposed or made aware of more opportunities for people with a physical disability once they were part of a community of people with physical disabilities. It was much easier for them to receive guidance from such communities with regards to finding employment, learner ships, access to higher level education, etc. Prior to that, finding opportunities for active productive participation was difficult.

“Let's say you’re going to be a packer, I won’t be able to pack things because the place is too small and even I can go there by the town by the mall, they have my qualifications, but they will just look at my wheelchair and say, ‘you won’t be able to do this because of your chair’. Even now I’m lucky I got a learner ship. But I did talk to some of my coaches, I talked to them and said I want to come to Johannesburg and they said they could make a plan, I could ask my teammates if they can get me something on the other side because there's a lot of opportunities in Joburg. Here there is less opportunity for people like us. So, if I let them help me then my situation will change from where I am.”—AX1000

“While I was at Eros school, we went to a career exhibition in Northlink, so there was CPUT, there was UCT, Stellenbosch and UWC. So, I always wanted to go to Stellenbosch 45 https://scholar.sun.ac.za but I thought it was in town I don’t know why. The reason why I wanted Stellenbosch because of their sports programs so that's why I wanted to go there. So, after the career exhibition I met this lady who gave me her email address and she assisted me with my application.”—NK2000

“..the first time I experienced basketball was now while I was in rehab because we played a few games in rehab. That was my first-time watching people play basketball and now I'm trying it. I got a chance to meet a lot of people and being around other wheelchair users, you feel freer compared to being around people who don’t use wheelchairs. It was recommended at the rehab, I was given the contact details for the people who help with disabilities, so when I got discharged from rehab I was already talking to those people. I was put in contact with QASA whilst I was in rehab and even for me to admitted to False Bay, I was recommended via email to False Bay by the rehab.”—SM1000

Theme 3: Improved self-esteem and self-confidence

The final theme focused on how participants expressed how participation in wheelchair basketball helped them to improve their self-confidence and self-esteem through improved social support from both family and friends and by assuming the perceived role of being a community role model. Collectively this helped with reducing episodes of self-isolation and improved recognition of sense of purpose amongst participants, especially when they observed how they were perceived by society through their assumed community role model positions physically and through social media.

Sub-theme A: Decreased self-isolation and improved sense of purpose

Most of the participants expressed that after their injury they initially felt they would not be able to do anything for themselves without needing some form of assistance from family and friends. They would participate less in activities because they felt that they were a burden by continuously needing assistance. Therefore, they would remain in the comfort of their homes and only go out when needed. However, once they gained the skills and physical independence, they required less assistance and felt like less of a burden which then improved their self-isolation and with that they were able to explore and experience different things in their lives that gave them a sense of purpose.

“Uhm, what can I say.. It gave me a motive that there's, that I can put myself in that place that I want to be like the others”—AX1000

“Before I was always alone at home, so I was not even getting used to the people because of myself. It made it better when I started to learn to accept myself the way I am”—MM200

“As I said before I was insecure and I was struggling to find myself and my purpose in life so it helped me a lot. I think since I'm growing, I find things more flexible and I think I am more independent Also joining the national team, I realized that anything is possible if you just believe in yourself and don’t stay in you comfort zone of “I'm disabled, I cannot do stuff”.—NK2000

“I think it's better, it's not the same as before because now I go outside more, I don’t stay inside like I used too in 2017/2018. It's really not the same, there's been a big difference. I only ever left the house if there was somewhere I needed to go”—SM1000

Sub-theme B: Community role model

With a gain in functional independence and self-confidence over the years. With ongoing encounters with peer supporters, the participants slowly felt comfortable with assuming being role models in their own community.

“There's nothing much I can say. I have Facebook, I have Instagram so sometimes when I'm travelling with the national team I share my pictures on Facebook and Instagram so they support me and tell me we are behind you all the way” and then sometimes if I'm wearing my Sasol shirt they will be like ‘our hero’. It's almost like I'm their leader in the community because I have travelled more unlike them.”—NK2000

“It's because in some parts like when I see a child in a wheelchair, it reminds me of way back when I was in school and people weren’t getting used to me and pointing out that I’m in a wheelchair and it affected my life. So, when I see a child especially in a wheelchair I feel like I can help the child”—MM2000

Sub-theme C: Improved social support from friends and family

As much as rehabilitation is aimed at improving functional independence, it does not take away from a need for personnel assistance. Building and maintaining relationships is an important part of the rehabilitation process. All the participants expressed some sort of support throughout their lives from either family, friends and/or community. The social support improved even further once family or friends were also acquainted with other family members and friends of members of the wheelchair basketball community. This created a supportive environment not only the participants but also for their family and friends.

“…they support anything that has to do with my wheelchair. It started on the first days I went to Joburg when I played in the SuperSport Series for the first time and they watched me on TV and they are starting to understand wheelchair basketball from watching it and supporting me.”—SM1000

“..they are very good especially my family because they help me in everything, I don’t feel like I’m not welcome when I’m around my community and my family”—NK2000

“Family I would say they support me sometimes. Sometimes it's better, sometimes it's lacking. And with friends, basically my friends always push me and say to keep going further.”—MM2000

### 3.3 Integration of questionnaire and interview findings

Triangulation of quantitative and qualitative data revealed important patterns. Participants with higher CIM scores (perceived integration) generally described stronger peer support networks in interviews. In comparison, those with lower CIQ productivity subscale scores described significant employment and educational barriers in their narratives.

For example, the participant with the highest CIM score described extensive peer support, stating: “Being around other wheelchair users, you feel freer.” Conversely, the participant with the lowest CIQ productivity score reported: “Here there is less opportunity for people like us.”

The discrepancy between higher perceived integration and lower objective integration suggests that wheelchair basketball may enhance subjective feelings of belonging even when structural barriers to full community participation persist. This interpretation is supported by participant narratives emphasising psychosocial benefits despite ongoing environmental challenges.

## 4 Discussion

This study focused on contributing to current knowledge on community integration of people with spinal cord injuries by exploring the role of participating in wheelchair basketball on enhancement of community integration. In this discussion, a cross critical analysis of the findings is construed and discussed with reference to the themes, key objectives, and current literature. The observations and insights of the researcher are also carefully integrated throughout the discussion.

### 4.1 How participating in wheelchair basketball facilitates continuous rehabilitation in the community

This study revealed that wheelchair basketball participation enhanced functional independence through three mechanisms: improved physical fitness, acquisition of wheelchair skills, and peer learning. The demanding nature of wheelchair basketball makes the participants physically fitter than the general SCI population. This is consistent with findings by Van Der Westhuizen (10), that 85.73% of people with an SCI, specifically physically fit individuals with paraplegia, report satisfaction with their quality of life due to only mild to moderate participation restrictions. Participants navigate environmental barriers more effectively after joining the team and demonstrated the ability to perform high-level transfers, manoeuvre over uneven terrain, and navigate inaccessible areas, skills learned primarily from senior teammates rather than through formal rehabilitation. This peer-to-peer skill transfer represents an important mechanism through which team sports extend rehabilitation into community settings. This principle also applied to accessing educational facilities and/or opportunities and these findings confirm this principle.

Regarding secondary complications, three of four participants reported reduced complications post-participation. This finding aligns with the literature, which identifies secondary complications as a major barrier to community participation. In a study by Vermaak et al. (11), secondary complications such as, bladder infections, pressure sores and pain contributed to 17% of what people with SCI considered as a personal barrier. These secondary health conditions were the main cause of re-hospitalizations which resulted in prolonged bed rest and subsequent physical inactivity (11). Most of the participants did not report any history of secondary complications and re-hospitalization since they participated in wheelchair basketball, and it was mostly attributed to an increase in physical activity and increased participation in their community.. Therefore, recreational activities such as wheelchair basketball alongside rehabilitation strategies need to be implemented to help reverse this pattern of physical inactivity (12).

### 4.2 Social support systems and self-efficacy provided through participating in wheelchair basketball

According to Hitzig et al. (13), people with a SCI are often faced with a number of challenges in developing and maintaining social relationships and community participation resulting in major physical and psychological changes In the study, we explored the level of community integration of participants subjectively and objectively by using the CIM and CIQ, respectively. The discrepancy between higher CIM scores and lower CIQ scores is particularly noteworthy. This finding suggests that, while participants perceive themselves as well-integrated, likely due to strong social connections and peer support in wheelchair basketball, objective barriers to full community participation persist, particularly in home independence and productive activities. This aligns with participant narratives describing ongoing environmental barriers despite improved confidence and skills. Previous studies have found similar patterns, where subjective integration measures often exceed objective indicators in disability populations, reflecting adaptation and changed expectations rather than actual environmental accessibility.

The study explored participants' levels of community integration, both subjectively and objectively. Questionnaire results showed that participants with higher perceived integration after wheelchair basketball exhibited lower levels of self-isolation. This occurred alongside stronger support systems before and after becoming team members.

The presence of family and friends also provided better social support, especially for participating in recreational activities. Participants noted that when family members and friends were exposed to the community of wheelchair users, they themselves gained exposure and subsequent support from other family members within that community. This provided greater understanding, through peer education and exposure, of the community of wheelchair users and people with spinal cord injury.

One of the main aspects expressed repeatedly by all participants was a notable improvement in their self-esteem and self-confidence when they became part of the wheelchair basketball community. The challenging nature of the sport fostered a sense of willingness to achieve more, and once they did, their self-confidence grew. This confirms previous studies by Hamrouni et al., (14) and Bozkuş (15) that found wheelchair basketball played a positive role in players' self-esteem development and better personal identity reconstruction which aids with reintegration into society because of the improved self-image and the newly acquired autonomy. It may therefore be the case that, participating in sports over time has the potential to bridge a gap between institutional rehabilitation and community re-integration by improving overall good physical function, low rate of acquiring secondary complications and maintaining good mental health and psychosocial support.

### 4.3 How do team sports for people with an SCI enhance re-integration and participation into their own community?

While some studies such as, Urbański et al. (16) who stated that individuals with SCI who participate in team sports and those that participate in individual sports experience similar degrees of re-integration suggesting similar reintegration outcomes between team and individual sport participants, our findings suggest that team sports may offer unique advantages during early reintegration stages. The participant who was exposed to wheelchair basketball during inpatient rehabilitation noted that earlier involvement might have accelerated his recovery. Conversely, a longer-term participant had recently begun exploring individual sports after building confidence through team participation.

This pattern suggests a developmental trajectory: team sports may provide essential psychosocial support and peer learning during initial community reintegration, after which individuals may feel confident pursuing individual activities. This has implications for rehabilitation programming, suggesting that exposure to team sports should occur early in the reintegration This is further supported by Anneken et al. (17) who stated that exposure and participation in sports in the rehabilitation phase may contribute to the improvement of rehabilitation outcomes and provide valuable skills needed for re-integration, including assistance in maintaining physical and mental health as well as coping with daily stressors.

### 4.4 Reflections from observations and journaling

During data collection, participants were observed during training sessions and official games. These observations allowed for the observation of interactions between participants, both with their immediate teammates and with their opponents. One thing that stood out during these observations was the change in confidence depending on the environment. Participants exhibited more extroverted and comfortable behaviour around their own teammates than around unfamiliar people, such as non-disabled spectators.

The types of conversations among participants were generally about their daily activities and family life. Among male participants, conversation usually revolved around the last game they played or watched on television, as well as tips and tricks regarding their home exercise routines. The atmosphere is very relaxed, and participants seem comfortable in their environment.

### 4.5 Conclusion

Prior to participation in wheelchair basketball, the participants expressed a low level of community re integration. All of them experienced phases of poor social support, self-isolation, and they showed a lack of interest in engaging with their greater community. Being part of a wheelchair basketball team has had a positive influence on all the participants and their respective families and friends in different aspects of their lives. Improvement in their physical independence meant feeling less of a burden to their family. This further translated to improved self-confidence, provided a general sense of well-being. this improved the feeling of inclusivity and increased socialization created awareness and opened opportunities for the participants. The ability for the sport to address multiple components in order to achieve a common goal of successful community integration makes it a viable tool that can be used and this was the overall experience of the participants.

## 5 Recommendations and proposed future interventions

Based on the current findings of the study and analysis of the results, the following recommendations are presented to improve participation of people with a spinal cord injury in wheelchair basketball:

1. Revision of the South African health policies to adequately define and strengthen the role of rehabilitation in the health sector including the long-term interventions in the communities. This could be done by shifting the rehabilitation role away from primary healthcare centres (where medical needs take priority) and more into community participation and involvement.
2. The Department of Health needs to re-evaluate funding and resource allocation, specifically pertaining to the support of rehabilitation facilities including community-based rehabilitation centres and people with disabilities overall. This can be achieved by providing and/or allocating more resources towards sports and recreational activities to health and rehabilitation facilities. In conjunction with the health department, the department of Sport and Recreation can provide support to local sports clubs in a form of transportation, accessibility to safe facilities, provision of equipment, fundraising and/or providing financial resources and creating awareness and youth developmental programmes. The key is to make participation opportunities accessible for people with a disability, including SCIs.

Future research could focus on more clubs and possibly include other types of impairments within team sports to understand the different roles played by team sports in their rehabilitation process.

## 6 Limitations of the study

### 6.1 Sample size

The sample size for this study is very small due to the focus on one health condition instead of considering the different variations of health conditions that are present in a wheelchair basketball team. Post-COVID19, many of the wheelchair basketball clubs in the Western Cape region were unable re-open and fully function again, therefore, there was only one local club that could provide suitable participants. School clubs were not considered due to the age limitations of the study in the inclusion/exclusion criteria.

### 6.2 Geographical location of the study

This study only focuses on the Western Cape region and not a true reflection of South Africa as a whole. If certain provinces such as the Mpumalanga province, which does not have specialized SCI or rehabilitation units in the public sector, were taken into consideration, what impact would that have had on the result of the findings?

### 6.3 Type of sport

There are a number of different adaptive sports that cater to people with a SCI, can the type of sport influence the level of participation based on interest? As the research in this study has shown, the type of sport and whether an individual participated in sports premorbid, may have an impact on their interest and motivation to participate in sport post injury.

## Data Availability

The raw data supporting the conclusions of this article will be made available by the authors, without undue reservation.
